# Speech intelligibility with and without noise in individuals exposed to electronic music

**DOI:** 10.1590/S1808-86942010000300002

**Published:** 2015-10-20

**Authors:** Jéssica Kuchar, Cássia Menin Cabrini Junqueira

**Affiliations:** 1 Specialist in Clinical and Educational Audiology -Craniofacial Anomalies Rehabilitation Hospital – HRAC from the University of São Paulo – USP. Speech and Hearing Therapist – Foundation for the Study and Treatment of Craniofacial Anomalies – FUNCRAF; 2 MSc. in Communication Disorders – Tuiuti University of Paraná. Professor of the Hearing and Speech Program – Maringá University – CESUMAR. Centro Universitário de Maringá – CESUMAR

**Keywords:** audiometry, noise, speech

## Abstract

Audiometry is the main way with which hearing is evaluated, because it is a universal and standardized test. Speech tests are difficult to standardize due to the variables involved, their performance in the presence of competitive noise is of great importance.

**Aim:** To characterize speech intelligibility in silence and in competitive noise from individuals exposed to electronically amplified music.

**Material and Method:** It was performed with 20 university students who presented normal hearing thresholds. The speech recognition rate (SRR) was performed after fourteen hours of sound rest after the exposure to electronically amplified music and once again after sound rest, being studied in three stages: without competitive noise, in the presence of Babble-type competitive noise, in monotic listening, in signal/noise ratio of +5 dB and with the signal/noise ratio of 5 dB.

**Results:** There was greater damage in the SRR after exposure to the music and with competitive noise, and as the signal/noise ratio decreases, the performance of individuals in the test also decreased.

**Conclusion:** The inclusion of competitive noise in the speech tests in the audiological routine is important, because it represents the real disadvantage experienced by individuals in daily listening.

## INTRODUCTION

For some decades now, noise has been the subject of studies regarding hearing loss prevention programs in workers; however, we know that noise does not occur exclusively in the workplace, it is present in all environments where we live: home, leisure, sports, school^1^.

The human ear is sensitive to a variety of acoustic activities. Noise, in general, is mentioned by several authors as cause of physiological and psychological changes on the individuals exposed to it^1^, ^2^, ^3^, ^4^. One of the auditory effects it causes to the body is interfering in oral communication produced by the masking caused by background noise from speech sounds, although its main effect is the loss of hearing^3^.

When a conversation happens in noisy places, it requires double attention from the individual who is conveying the message, because the skill to understand speech in this situation is reduced. Gama^5^ says that speech recognition is accompanied by the combination of acoustic, linguistic, semantic and circumstantial cues. When listening occurs under favorable conditions, the clues are present in excess, and some can be disregarded^5^. For an effective message transmission, there is a redundancy of acoustic cues which the listener uses according to the communication context and situation^5^.

However, when listening in noisy environments, there is a decrease of acoustic cues in the message, leading the listener to use other clues to understand it. It is therefore important that the audiological evaluation have methods to evaluate the real disadvantage of the individual in situations of unfavorable listening, thus reproducing everyday listening.

Therefore, this study sought to characterize the possible consequences of noise in the speech intelligibility of individuals exposed to electronically amplified music.

## MATERIALS AND METHODS

This study was conducted in a Speech Therapy Clinic of a higher education institution located in northern Paraná, obtaining the assent of the Ethics and Research Committee of this institution under protocol number 241/2005.

20 college students participated in this study, people with hearing thresholds within the normal range, type A tympanometric curve and acoustic reflex between 70 and 100 dB; 10 females and 10 males, aged between 18 and 25 years, the average age being 20.25 years, they all signed a consent form.

The material used was: a multiple questions questionnaire, a HEINE otoscope, supra-aural earphone TDH-39, B-71 bone vibrator, Interacoustic audiometer, model AC 40, Interacoustic immitancemeter, model AZ 7 Discman, CD with a recording list of monosyllables and babble noise. The audiometer, headphones and bone vibrator were calibrated, according with ANSI 53.6 – 1996 / ISO 389 to 1991 / ISO 8798 / ANSI 53.43 – 1993 Standards.

We first inspected the external acoustic meatuses of the individuals in order to guarantee that the audiological evaluations were carried out in favorable conditions. Next, a questionnaire was applied (Table 1), in order to obtain information regarding the hearing health of individuals.

**Table 1:** Results regarding the questionnaire pertaining to hearing health

Question	Yes	%	No	%
1. Family history	6	30%	14	70%
2. Middle ear disorders	9	45%	11	55%
3. Exposure to intense noise	5	25%	15	75%
4. Habit of listening to loud music/TV	13	65%	7	35%
5. Irritability in noisy environments	6	30%	14	70%
6. Headache	10	50%	10	50%
7. Vertigo and/or dizziness	2	10%	18	90%
8. Nausea	0	0%	20	100%
9. A sensation of clogged ear	4	20%	16	80%
10. Tinnitus	4	20%	16	80%
11. Reports good hearing	19	95%	1	5%

Audiological assessment was initiated by acoustic impedance measurements (dynamic and static compliance and acoustic reflex thresholds ipsilateral and contralateral) and pure tone audiometry thresholds where the air conduction was investigated in the frequencies of 250, 500, 1000, 2000, 3000, 4000, 6000, 8000 Hz and bone conduction at frequencies of 250, 500, 1000, 2000, 3000 and 4000 Hz, and the criteria used for classification of audiometric curves followed the BIAP No. 02 / 1 bis (1996) recommendation^6^.

Then, we did logoaudiometry: speech recognition threshold (SRT) and speech recognition percentage index (SDT), which is done in three stages: the first with no competitive noise, the second with the babble type competing noise in monotic hearing, in the signal to noise ratio (S / R) of + 5 dB and the third well on these criteria, but with the S / N ratio of – 5 dB, as proposed by Kumabe^7^.

It is worth mentioning that the SRPI was done at three different times. The first time it was carried out before the individual was exposed to electronically amplified sound, after fourteen hours of sound rest; the second time happened immediately after exposure and the third moment was once again after fourteen hours of sound rest.

The subjects were exposed to electronically amplified music, at an intensity of 100dB HL in a soundproof booth, using a Discman coupled to an audiometer, during 30 minutes, and the sound output was in the TDH-39 phones, for greater reliability on the material. For statistical analysis of the moments before and after exposure to music in every situation: without competitive noise, with competitive noise in S/R +5dB and −5dB we used the signal test8 through the statistical package Statistica 7.1 (Statsoft).

## RESULTS

### Questionnaire

Table 1 depicts the main data obtained from the answers given in the questionnaire deployed to the individuals who participated in this study.

External Auditory Canal Inspection

The results obtained through inspection of the external auditory canal showed that the individuals surveyed did not have any change that prevented audiological evaluations from being conducted under favorable conditions.

### Immittance testing

Regarding acoustic immittance measures, we did not find changes. All subjects had type A tympanometric curves bilaterally and contra and ipsilateral afferent and efferent stapedial reflex in both ears.

### Tonal Hearing Thresholds

All subjects evaluated had hearing thresholds within normal limits, lower than 20dBHL in the frequencies of 250 to 8,000 Hz, according to BIAP6 recommendation.

### Percentage Index of Speech Recognition (SDT)

Tables 2, 3 and 4 show the SDT, performed in silence and in the presence of noise in S/R of +5 dB and −5 dB for all subjects in the study before and after exposure to electronically amplified music at 100 dB of intensity, through the TDH-39 phones in a soundproof booth, respectively.

**Table 2:** SDT done before and after exposure to electronically amplified music without competitive noise.

	RE		LE	
	Before	After	Before	After
Subject 1	100%	100%	92%	92%
Subject 2	100%	100%	96%	96%
Subject 3	100%	100%	100%	100%
Subject 4	100%	96%	100%	96%
Subject 5	100%	96%	100%	100%
Subject 6	100%	100%	100%	100%
Subject 7	100%	100%	100%	100%
Subject 8	100%	100%	100%	100%
Subject 9	100%	100%	100%	100%
Subject 10	100%	100%	100%	100%
Subject 11	100%	100%	100%	100%
Subject 12	100%	100%	100%	100%
Subject 13	100%	100%	100%	100%
Subject 14	100%	96%	100%	96%
Subject 15	100%	96%	100%	100%
Subject 16	100%	92%	100%	100%
Subject 17	100%	100%	100%	100%
Subject 18	100%	100%	100%	100%
Subject 19	100%	96%	100%	100%
Subject 20	100%	96%	100%	100%

**Table 3:** SDT done before and after exposure to electronically amplified music in the +5dB signal to noise ratio.

	RE		LE	
	Before	After	Before	After
Subject 1	100%	96%	96%	92%
Subject 2	100%	100%	92%	96%
Subject 3	100%	100%	100%	96%
Subject 4	100%	100%	92%	92%
Subject 5	96%	100%	92%	96%
Subject 6	100%	100%	100%	96%
Subject 7	100%	100%	88%	92%
Subject 8	100%	96%	100%	96%
Subject 9	100%	96%	100%	92%
Subject 10	100%	92%	92%	88%
Subject 11	100%	100%	100%	96%
Subject 12	96%	96%	100%	96%
Subject 13	100%	100%	92%	92%
Subject 14	100%	96%	100%	96%
Subject 15	100%	100%	88%	96%
Subject 16	96%	100%	100%	100%
Subject 17	100%	100%	100%	100%
Subject 18	96%	96%	88%	88%
Subject 19	100%	96%	96%	96%
Subject 20	100%	100%	100%	100%

**Table 4:** SDT done before and after exposure to electronically amplified music in the −5dB N/S ratio.

	RE		LE	
	Before	After	Before	After
Subject 1	68%	72%	88%	72%
Subject 2	64%	68%	64%	60%
Subject 3	60%	64%	84%	72%
Subject 4	68%	68%	64%	60%
Subject 5	68%	68%	60%	52%
Subject 6	84%	84%	84%	68%
Subject 7	80%	80%	68%	60%
Subject 8	84%	80%	76%	48%
Subject 9	64%	64%	68%	52%
Subject 10	56%	52%	64%	68%
Subject 11	76%	68%	92%	72%
Subject 12	52%	64%	64%	64%
Subject 13	72%	68%	76%	56%
Subject 14	68%	68%	72%	40%
Subject 15	60%	56%	56%	60%
Subject 16	56%	56%	84%	68%
Subject 17	88%	88%	80%	72%
Subject 18	60%	60%	76%	68%
Subject 19	52%	44%	72%	80%
Subject 20	80%	72%	72%	60%

In the SDT before exposure and without competitive noise, the individuals had a mean score of 100% before exposure and 99% afterwards, while the evaluation in noise at S/N Ratio +5dB the subjects had an average of 98% correct answers before exposure and 97% after.

By analyzing Table 4, again we found that speech intelligibility is impaired in the presence of competitive noise, considering the lower S/N ratio, we found the values in percent for speech recognition in the S/N – 5 dB i.e., noise 5 dB more intense than the speech signal. Mean SDT in this relationship were 71% correct before exposure to 100dB of electronically amplified music and 65% after such exposure. One can see the difference between the values, when comparing the test conducted in the S/N at −5 dB with the same carried out without background noise and the SNR of +5 dB.

**Figure 1:**
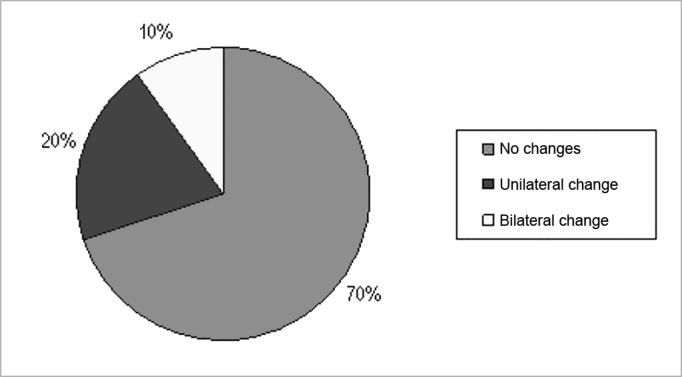
Percentage of SDT alterations which happened after exposure to music without competitive noise.

**Figure 2:**
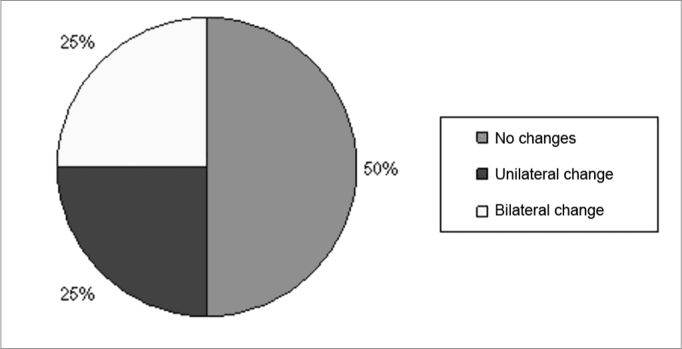
Percentage of SDT alterations which happened after exposure to music in the + 5dB S/N ratio.

Figure 1 shows the percentage of individuals who had changes in SDT after exposure to music without competitive noise. The Signal Test showed an SDT without competitive noise before and after exposure to electronically amplified music, statistically significant difference only for the right ear (p = 0.023), which was the first tested ear. Figure 2 shows the percentage of individuals who had changes to the SDT in the presence of background noise in the SNR of +5 dB. In Figure 2 one may notice that 50% of patients showed abnormalities in SDT with competitive noise in the SNR of +5 dB, and 25% of those with unilateral and 25% with bilateral involvement. It was also noted that 50% of the subjects showed no change in SDT after exposure to electronically amplified music.

The Signal Test in SDT with competitive noise in the SNR of +5 before and after exposure to electronically amplified music showed no statistically significant difference for the ears (p > 0.05).

**Figure 3:**
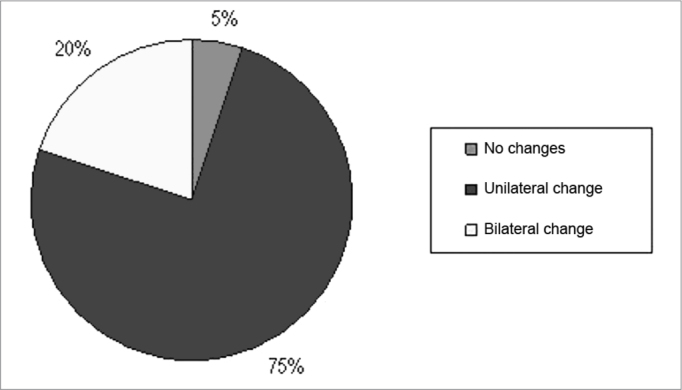
Percentage of SDI alterations which happened after exposure to music in the −5dB S/N ratio.

Figure 3 depicts data, in percentage of individuals who had changes in SDT performed with competitive noise in the S/N of −5 dB. Note that only 1 subject (5%) did not show any change in speech intelligibility after exposure to electronically amplified music in the SNR of −5dB; 4 (20%) had bilateral involvement, or had the two ears worse after exposure to music and 15 subjects (75%) showed alterations in one ear. Therefore, we noticed that, considering the mean total bilateral values, there are really big changes in this S/N ratio.

**Figure 4:**
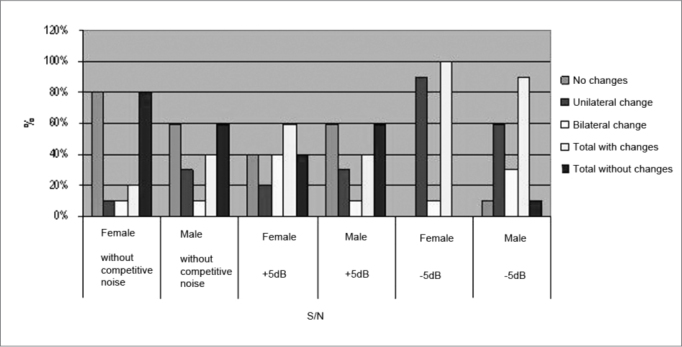
Percentage of SDT alterations which happened after exposure to music broken down by gender in the different S/N ratios used.

The Signal Test in SDT with competitive noise in the S/N of −5 before and after exposure to electronically amplified music showed a statistically significant difference both for the right ear (p = 0.039) and for the left ear (p = 0.006). Figure 4 shows the occurrence of changes after exposure to electronically amplified music, by gender, without competitive noise in the presence of background noise in the SNR +5dB and SNR −5dB. It also shows the total changes for both genders in all test conditions used.

To compare genders we only calculated the arithmetic mean for the groups and compared them. Without the presence of competitive noise, females performed better than males, because 8 subjects (80%) showed no change in the face of 6 male subjects (60%). For females under this test condition, 1 subject was found with unilateral and 1 with bilateral involvement, and in males, 3 with unilateral and 1 with bilateral involvement.

In the second test condition, i.e., in noise at SNR at +5 dB, males had the best performance, with 6 subjects (60%) showing no change in speech intelligibility, compared to 4 female subjects (40%). Of the changes found in women, 4 individuals (40%) showed bilateral involvement and 2 (20%) a unilateral change; in males, there were 3 individuals (30%) with changes in one ear and 1 individual (10%) with changes in both. Finally, for testing with competitive noise in the S/N of −5dB, females had worst performance again, with 100% of individuals with alterations after exposure to electronically amplified music, these being 90% of unilateral change and 10% bilateral involvement. In males, we noticed 90% of individuals with some type of change, 60% unilateral and 30% bilateral.

The SDT was performed for the third time after 14 hours of acoustic rest in order to confirm the return of the hearing thresholds of patients and, consequently, their SDT. We noticed that all subjects had 100% SDT in both ears in the third assessment, without competitive noise, confirming the ease of performing the test in silence. In implementing the same in the presence of noise with SNR of +5 dB, one can see that only 2 individuals had the SDT lower than that of the first application of the test. Finally at the completion of SDT in the S/N of −5dB at the third application of the test, we noticed 6 subjects with low test scores, showing deterioration in speech recognition.

## DISCUSSION

According to Hall (1999)^9^, to classify as normal hearing, individuals should be inspected audiologically and otologically, not have a history of middle ear pathologies or exposure to ototoxic medications, or exposure to excessive noise and no complaints of tinnitus. According to the author these strict prerequisites would not be found in most individuals with hearing thresholds < 20dB HL.

All subjects in the present study, after exposure to electronically amplified music, had thresholds in the frequencies of speech preserved and drops on the high frequencies because with the study we intended to provoke a TTS (“Temporary Threshold Shift”) on the individuals, to check for loss of speech intelligibility after exposure to electronically amplified music. This type of hearing loss is characterized by a reduction in hearing sensitivity, affecting mainly the high frequencies from 2,000Hz to 6, 000Hz^2^,^3^. Table 2 shows the values obtained from the SDT without competitive noise before and after exposure to music electronically amplified. This step had the highest number of correct answers in the three procedures performed, and all individuals studied showed SDT higher than 92%. Similar results were found by Caporali Silva^10^ in their studies, but with noise (“cocktail party”) and different populations.

Fletcher (1953) cited by Schochat^11^ investigated the relationship between speech energy and intelligibility, noting that high frequencies contribute with 60% for speech intelligibility and 5% for speech energy, while in the low frequency energy concentration is highest and intelligibility the lowest; therefore what also interferes with speech recognition are the high frequencies, which revealed the present study, since the intelligibility of speech was impaired even without lowering low frequencies. With respect to the SDT performed in the presence of competing babble-type noise in S/N ratio of +5dB (Table 3), you may notice a worsening of the results even when compared with the test without competitive noise before and after exposure to electronically amplified music. Although the changes in speech intelligibility found are not very significant, note that there was a change in these two criteria used to determine possible changes in speech intelligibility of subjects, such as the presence of a competitive noise and exposure to high levels of sound pressure.

Several authors have pointed the importance of the S/N ratio used in speech tests, and the higher this ratio, i.e. speech higher than noise, the better the individual's speech intelligibility^12^, ^13^, ^14^, ^15^. In the literature there is disagreement among authors as to the different S/N ratios to be used in speech tests in the presence of competitive noise. For example, Costa^12^ reports that the ideal SNR depends greatly on the material to be used, and values between +5 and +12 would be well suited for working with monosyllables and masking with speech sounds. For Schochat and Pereira^13^, this ratio can vary from −10 to +20 dB. Costa, Iorio Albernaz^16^ after doing a study aiming at standardizing a speech test with lists of sentences in noise with normal subjects, estimated the SNR of −11 dB for individuals to recognize 50% of speech stimuli.

In the literature, we found no studies relating to speech intelligibility tests comparing females and males, just as in this study only comparisons between young and elderly individuals were carried out, including individuals with hearing loss and normal ones, comparing the different types of NIHL among others^10^,^13^,^17^. However, this study showed that males performed better on tests in the presence of background noise in both S/N ratios used, while females had better performance when the same test was done without the presence of noise. Mantelatto and Silva^14^reported that the increased level of noise causes a greater effort to identify words; it is likely that cognitive processes and memory are involved in this task. They add that in a difficult situation of listening, speech hearing shall be determined by factors other than the acoustic signal, among them analysis of the context (phrases), expectations of the listener, attention resources and memory components.

Speech recognition in noise can be seen as a task that demands memory usage and selective attention, because the listener needs to focus attention on the message and store information in memory for speech, while ignoring the irrelevant information^10^. But during the test, possible interference with the individual attention may occur, compromising performance or increasing the variability of results: anxiety before a test situation, the exhaustion of individuals or any physical discomfort felt by them; and false positive responses, where the individual responds correctly by mental supply^16^.

The last application of the test after 14 hours of sound rest, one notices that some individuals had a decrease in the results, which may have occurred because of the factors previously described. Nonetheless, they showed a better ear, confirming a learning effect due to the memory used to record the word that was said and repeat it. Therefore, the more times the test is performed in a given period of time, the better the individual is bound to perform.

## CONCLUSION

Based on the data obtained, we can conclude that as the signal to noise ratio (SNR) decreases, individuals worsen their performance on speech intelligibility, and this performance tends to deteriorate further after they are exposed to high sound pressure levels.

We can also notice from this study that females had a better performance on the SDT without competitive noise, and males had better performance on the SDT in the presence of background noise in both S/N ratios used: +5 and −5 dB.

There was also a learning effect of the subjects in the 3rd test application, since all had better performance, since the lists were repeated several times.

It is noteworthy the importance of further studies involving the use of background noise in speech tests in subjects exposed to high levels of sound pressure, because this represents a real disadvantage experienced by individuals in everyday listening situations.
